# Myofibril Changes in the Copepod *Pseudodiaptomus marinus* Exposed to Haline and Thermal Stresses

**DOI:** 10.1371/journal.pone.0164770

**Published:** 2016-11-08

**Authors:** Ali Ibrahim, Anissa Souissi, Aymeric Leray, Laurent Héliot, Bernard Vandenbunder, Sami Souissi

**Affiliations:** 1 Interdisciplinary Research Institute, USR 3078 CNRS, University of Lille –Parc scientifique de la Haute Borne, 59650, Villeneuve d'Ascq, France; 2 Univ. Lille, CNRS, Univ. Littoral Cote d’Opale, UMR 8187 LOG, Laboratoire d’Océanologie et de Géosciences, F-62930, Wimereux, France; Helmholtz-Zentrum fur Ozeanforschung Kiel, GERMANY

## Abstract

Copepods are small crustaceans capable to survive in various aquatic environments. Their responses to changes in different external factors such as salinity and temperature can be observed at different integration levels from copepod genes to copepod communities. Until now, no thorough observation of the temperature or salinity effect stresses on copepods has been done by optical microscopy. In this study, we used autofluorescence to visualize these effects on the morphology of the calanoid copepod *Pseudodiaptomus marinus* maintained during several generations in the laboratory at favorable and stable conditions of salinity (30 psu) and temperature (18°C). Four different stress experiments were conducted: at a sharp decrease in temperature (18 to 4°C), a moderate decrease in salinity (from 30 to 15 psu), a major decrease in salinity (from 30 to 0 psu), and finally a combined stress with a decrease in both temperature and salinity (from 18°C and 30 psu to 4°C and 0 psu). After these stresses, images acquired by confocal laser scanning microscopy (CLSM) revealed changes in copepod cuticle and muscle structure. Low salinity and/or temperature stresses affected both the detection of fluorescence emitted by muscle sarcomeres and the distance between them. In the remaining paper we will use the term sarcomeres to describe the elements located within sarcomeres and emitted autofluorescence signals. Quantitative study showed an increase in the average distance between two consecutive sarcomeres from 2.06 +/- 0.11 μm to 2.44 +/- 0.42 μm and 2.88 +/- 0.45μm after the exposure to major haline stress (18°C, 0 psu) and the combined stress (4°C, 0 psu), respectively. These stresses also caused cuticle cracks which often occurred at the same location, suggesting the cuticle as a sensitive area for osmoregulation. Our results suggest the use of cuticular and muscle autofluorescence as new biomarkers of stress detectable in formalin-preserved *P*. *marinus* individuals. Our label-free method can be easily applied to a large number of other copepod species or invertebrates with striated musculature.

## Introduction

Copepods are small crustaceans and a major component of zooplankton, which represents the main food for higher trophic levels in aquatic systems including fish larvae. Copepods occur in most aquatic environments, from large oceans to small ponds. They are even found in extended transient water so after heavy rains in wet plant detritus [1].

Copepod fitness can be influenced by environmental changes such as salinity and temperature. These changes could be due to seasonality, to the formation of a mixing zone between freshwater and salt water [2, 3], to anthropogenic reasons like coastal power plant stations [4], chemical contamination [5], or climatic changes such as global warming [6].

The abundance of copepods and their sensitivity to temperature and salinity fluctuations make these species good indicators for global warming [7]. Temperature is one of the main physical parameter that determines the spatial and temporal distribution of most copepods [8]. Independent of the optimal range of temperature as well as their degree of stenothermy (variance around the optimal temperature), copepods show sensitivity to thermal stress with different endpoints. A rich literature on the biology and ecology of copepods focuses on the effects of temperature on their development, growth, survival and reproduction [9–11]. Regarding their salinity tolerance, two evolutionary pathways are characterizing marine and freshwater copepods, even though copepods can be found in hypersaline habitats [12]. Among copepods, euryhaline species and particularly those living in estuaries have high physiological performances and are characterized by high osmoregulation capabilities [13]. However, when the environmental conditions are not optimal, these copepods experience stress that can be observed at different levels of organization from cells to individuals.

Studies at the molecular level revealed that copepod responses to physical or chemical stressors comprise the activation of a first and a second line of defense, which involves efflux protein pumps, detoxification enzymes, and chaperones [14]. The study of the effects of temperature and salinity fluctuations on both the development and the survival of the estuarine copepod *Eurytemora affinis* demonstrated that optimal conditions for naupliar survival and development are obtained at 15°C and 15psu, and that only extreme salinity conditions have a negative effect on the survival of this species [15]. These experiments highlighted the ability of copepods to survive environmental changes, and the high variability of copepod responses.

Because of their small size it is possible to examine the morphology of copepods in depth. Such a study could be done using Confocal Laser Scanning Microscopy (CLSM) considering their sensitivity to excitation by visible wavelength proven in the literature and in our study. However, most imaging studies of copepods with a confocal microscope were focussing on the morphology of adults [16] or on the identification of different stages of egg development [17] using lipophilic probes and fluorescent nucleic acid stains as markers of nuclei. Double labeling methods (Annexin or Tunel with propidium iodide) have allowed the detection of cell degradation before the death of nauplii produced by females fed on toxic diets [18]. In addition, non-linear microscopy techniques (i.e. second and third harmonic generation) have been recently used to detect apoptosis in whole-mount copepod nauplii exposed to different concentrations of nickel [19]. The autofluorescence properties of copepods have already been used for morphological studies but whether these properties can detect effects of environmental stressors remains uncertain.

In this paper we investigated the adult calanoid copepod *Pseudodiaptomus marinus* by using cell and tissue autofluorescence. Since endogenous autofluorescing compound are involved in metabolic (NAD(P)H, flavin, lipopigments) or structural (collagen, elastin …) functions at cell and tissue levels, autofluorescence could provide useful information on cell metabolism and differentiation [20]. On this basis, cell and tissue autofluorescence is increasingly applied to biomedical diagnostic. Confocal imaging of cuticular autofluorescence has for instance allowed high resolution visualization of the morphology of small crustaceans [21].

Until now no observations using optical microscopy were made on the effects of temperature or salinity fluctuations on copepods. To the best of our knowledge, only one study by CLSM on the grass shrimp *Palaemonetes pugi* has suggested that the loss of transparency upon salinity and temperature changes was due to the pooling of hemolymph in the tail, disrupting the uniformity of the refractive index in surrounding muscle fibers and increasing light scattering [22]. We therefore examined the effects of temperature and haline stresses by CLSM on the calanoid copepod *Pseudodiaptomus marinus* grown for several generations under favorable and stable conditions. This copepod species originated from Asia and has invaded during the last decades several estuarine and coastal areas in the northern hemisphere [23]. More recently, this species was observed in the Mediterranean Sea as well as in the North Sea [24, 25]. This species has a potential for invading large coastal ecosystems at global scale [11]. It is important to understand the physiological performances of this species and mainly its capacity to resist to several sources of stress from natural (i.e. salinity and temperature) and/or anthropogenic origins (i.e. pollution).

The main questions addressed in this study are (i) how long can copepods resist haline and/or thermal stresses and to which extent, (ii) what are the effects of these stressors on the morphological integrity of copepods and, (iii) how to quantify these effects using confocal laser scanning microscopy. Our goal was to determine new stress markers for these species and calanoid copepods in general, by using autofluorescence imaging.

## Materials and Methods

### A) Copepod preparation and manipulation

Individuals of *Pseudodiaptomus marinus* used in this study were initially sampled in May 2011 in Lake Faro (38°16'N, 15°38'E) in Sicily, Italy. Then they have been cultured continuously under favorable conditions. No permission was needed for this sampling of plankton. Individuals of *P*. *marinus* used here have not been previously stressed and were acclimated to the laboratory conditions during several generations (~2 years in the Marine Station of Wimereux, France). Several individuals were set aside from stock cultures run at a temperature of 18°C and a salinity of 30 psu and immediately fixed as control samples (C). In parallel, four different stressful treatments were applied to copepods taken from the same stock culture. Copepods were stressed by their immediate transfer from their initial culture condition (C) to a new medium at different temperatures and/or salinities.

Haline, thermal and mixed stresses were defined as follows:

1. A moderate haline stress (H15). The salinity was shifted from 30 to 15 psu and the temperature was maintained at 18°C.
2. A major haline stress (H0). The salinity was shifted from 30 to 0 psu and the temperature was maintained at 18°C.
3. A thermal stress (T). The salinity was maintained at 30 psu and the temperature was shifted from 18°C to 4°C.
4. A mixed stress (M). The salinity was shifted from 30 psu to 0 psu and the temperature was shifted from 18°C to 4°C.

As soon as a decrease or an arrest of the copepod swimming activity and a subsequent sinking was observed under each of these stresses, the experiment was stopped by adding 4% of buffered formalin. We targeted the same physiological state when individuals started to become comatose and measured the ‘time to succumb’ in each stress conditions [26].

### B) Copepod Imaging

Ten adult females of *P*. *marinus* from control and from each stressful treatment were imaged. They were sorted individually from the fixed samples, then analyzed and observed in a lateral position under a commercial confocal microscope (A1, Nikon) with a 40X water immersion objective (NA = 1.25). Most autofluorescing compounds are efficiently excited at wavelengths ranging between 350 and 600 nm [20]. Before developing our imaging protocol that was applied to all individuals, a preliminary spectral study of different regions of *P*. *marinus* adults revealed that the optimal autofluorescence excitation wavelengths were located between 405 nm and 561 nm. Consequently, we used three distinct laser excitation wavelengths for all subsequent copepod imaging: 405, 488 and 562 nm emitted from three distinct continuous wave laser sources adjusted with a power around 0.35 mW. The corresponding selected spectral emission bands were respectively: 425 to 475 nm, 500 to 550 nm and 570 to 620 nm. Pixel sizes were fixed to 0.31 μm in order to be able to correctly measure distances between muscle stripes (which were around 2 μm). The pixel dwell time and the micrograph size were 0.237 μs and 1024×1024 pixels, respectively. Three-dimensional reconstructions from a combination of multi stacked images were realized with the NIS Element software (Nikon V 4.00.01).

### C) Stripes analysis

**Fig 1:**
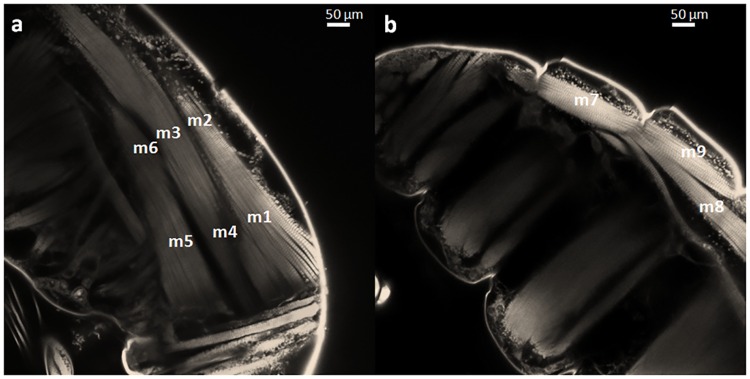
confocal laser scanning microscopy (CLSM) images of control *Pseudodiaptomus marinus* adult female prosome showing muscles regions m1 to m9. Bar = 50μm.

As shown in Fig 1, nine regions labeled as m1 to m6 in Fig 1a and m7 to m9 in Fig 1b, were defined in the prosome muscles viewed in lateral position. In each of these regions, intensity profiles were realized along myofibrils with Image J software (ImageJ 1.47q, http://rsbweb.nih.gov/ij/) and the sarcomere length was deduced from the average distance between five maxima (m lines). This measure was repeated five times in each muscle region.

The percentage of muscle fibers exhibiting stripes and the average sarcomere length measured for each muscle region of the 10 copepods used in control and different stress experiments were included in a database. From this database, average values were calculated and compared. A t-test was applied to test the significance of differences in the stripe size between control copepods and stressed copepods.

## Results

Copepods maintained at stable conditions (30 psu and 18°C) were subjected to instantaneous changes of temperature and/ or salinity and their movements along the different stressors were monitored visually. Immediately after their immersion in 15 psu water at 18°C (H15) copepods were moving normally; yet 3 hours and 30 minutes later some individuals began to sink. In contrast, immersion in 0 psu water at 18°C (H0) resulted in an immediate decrease in the swimming activity of copepods and comatose started after 12 minutes. The thermal stress (T) had milder effects; the shift from 18 to 4°C at constant salinity (30psu) slowed the copepod motion. This effect lasted for 75 minutes before most individuals began to become comatose. Finally, the mixed stress (M) of 0 psu and 4°C had the most drastic effects; copepods were immobile at these conditions and began to become comatose within less than one minute (Table 1). We subsequently investigated the morphological effects of these stresses.

**Table 1:** Physiological effects of the different stresses applied to copepods (temperature, salinity, duration).

Initial condition	Stress condition	Nature of stress	Symbol	Behavior at time 0 after stress	Stress application time
18°C, 30 psu	30 psu, 4°c	Thermal stress	T	Slow motion	75 minutes
18°C, 30 psu	15 psu, 18°c	Minor haline stress 15psu	H15	Motion	210 minutes
18°C, 30 psu	0 psu, 18°c	Major haline stress 0psu	H0	Minimal activity	12 minutes
18°C, 30 psu	0 psu, 4°c	Mixed stress	M	Immobility	Less than one minute

The detection of copepod autofluorescence by a confocal microscope allowed the visualization of the morphology of internal organs within 60 μm depth beneath the cuticle with a 0.31 micrometer spatial resolution with our acquisition parameters. The three excitation wavelengths available on the A1 Nikon confocal microscope enabled the observation of different types of fluorescence. The cuticle showed blue fluorescence (with a 405 nm excitation and emission between 425 and 475nm), whereas muscles fibers and lipid droplets showed green to red fluorescence (with a 488 and a 562 nm excitation wavelength and emission band, respectively, at 500 to 550 and 570 to 620 nm). These differences in the wavelength of emitted light reflect differences in the composition of these structures (Fig 1).

In control samples, the cuticle surrounding the copepod prosome was easily visualized: it had a thickness of 1 to 2 μm and it absorbed a great amount of the excitation light. The cuticle consists of several segments, each covering a segment of the copepod body. These fragments are interconnected via a flexible thin area allowing the movement of each fragment of the cuticle relative to the adjacent ones. This connection seemed to be more fragile and thinner than the fragments of the shell. A few micrometers in depth behind the carapace, the longitudinal muscles extended to the dorsal region of the copepod body. Transversal muscles in the ventral part were also observed (Fig 1). Individual muscle fibers with different sizes displayed similar levels of fluorescence, with stripes periodically ordered along their length in many regions of the prosome. Muscles were affected by haline and thermal stresses and the cuticle was affected by the major haline (H0) and mixed (M) stresses. No significant variation was observed after exposure to these stresses around the gut and in the appendages.

**Fig 2:**
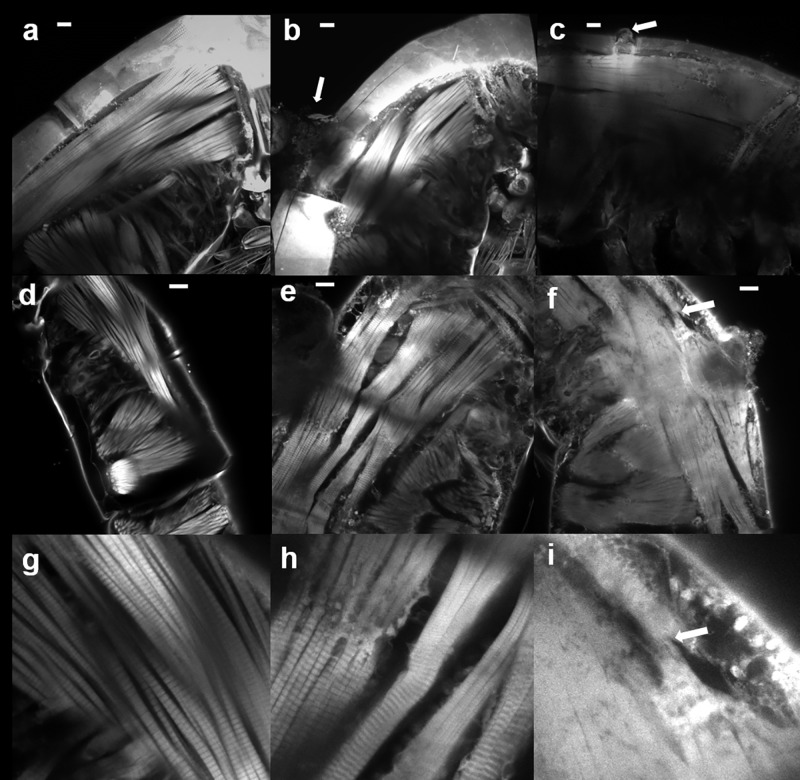
Effects of saline stresses on *Pseudodiaptomus marinus* female. a-c: 3D images of the upper body. a) control sample (C). b) mixed stress (M; 4°C and 0psu). c) major haline stress (H0; 4°C and 0 psu final). Arrows in b and c point to cracks in the cuticle and materials going outside. d-i: Optical sections illustrating the effects of different stresses on the cuticle and the longitudinal muscles, d & g) control samples with periodic stripes in muscle fibers and normal cuticle. e & h) mixed stress. Part of the muscles is stretched; and in the other part stripes are no longer distinguished. f & i) Major haline stress (H0) with the loss of the autofluorescence coming from muscles stripes but in some zones a cut in the muscle is observed (arrow). Bar = 20μm.

**Fig 3:**
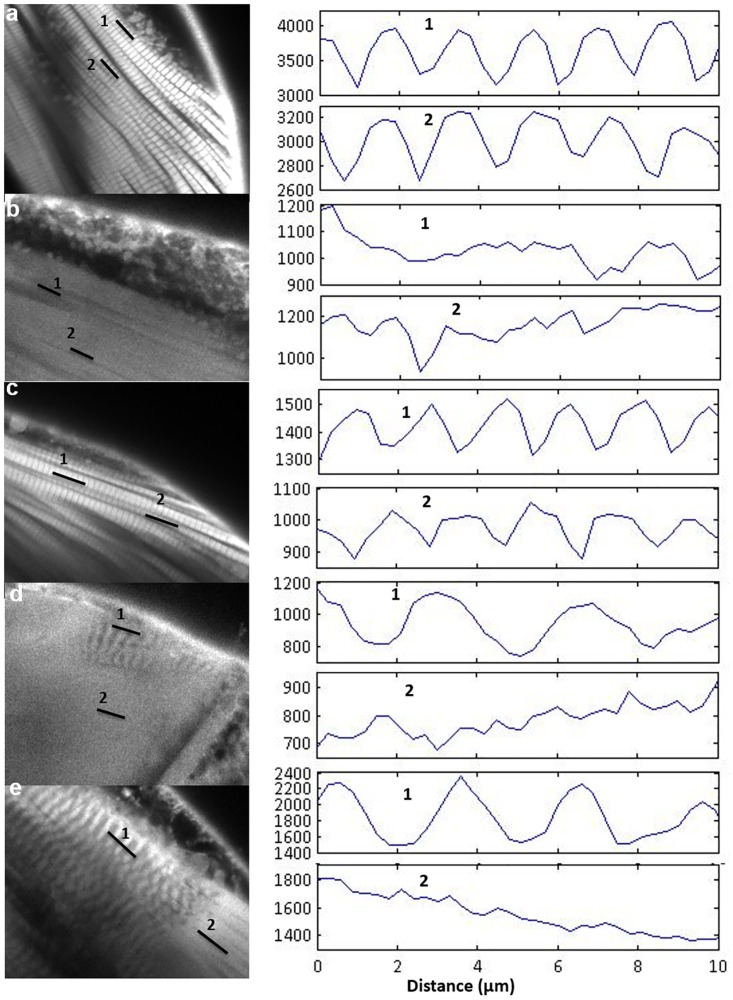
Analysis of the periodic distribution of muscle stripes after temperature or/and saline stresses with plot profiles of regions affected. a) control (C). b) minor haline stress (H15; 18°C, 15psu). c) Thermal stress (T; 4°C, 30psu). d) Major haline stress (H0;18°C, 0psu). e) Mixed stress (M;4°C, 0psu). Bar = 5μm.

**Fig 4:**
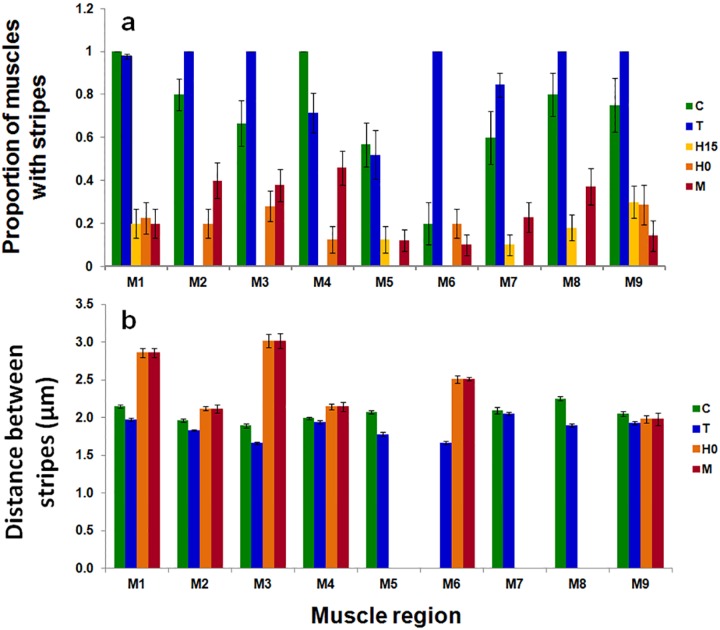
a) percentage of muscle fibers exhibiting stripes. b) Average distances between stripes in each muscle region and each stress. C, control (18°C, 30psu); H15, minor haline stress (18°C, 0psu, 12 min); H0, major haline stress (18°C, 15psu, 210 min); M, mixed stress (4°C, 0psu, less than one minute); T, thermal stress (4°C, 30psu, 75 minutes). Vertical bars correspond to standard errors.

The morphological changes caused by the four different stresses applied to *P*. *marinus* copepods were as follows:

A crack in the cuticle appeared after the major haline (H0) and the mixed (M) stresses in 7 out of 10 samples were examined at each condition (Fig 2b and 2c, arrows) but not observed in control samples (Fig 2a). This crack was always located between the cuticle of the first and second segment in the major haline stress (H0) and mixed stress (M), suggesting that it was the weakest point in the cuticle. In some copepods, material budding from the inner body from this crack or going outside was observed. In addition, haline stress resulted both in the disappearance of stripes from certain entire muscle regions (Figs 2f, 2i and 3d), compared to the control (Figs 2g, 2d and 3a) and abnormalities of these stripes (Fig 4). By looking at the mixed stress (M) in Figs 2e, 2h and 3e we noted a mix between stripe disappearance and stretched strips (the distance between stripes being increased compared to the control in Fig 3d, 3g and 3a.

These effects were analyzed and quantified by statistical analyses. At first, and shown in Fig 4 we studied the proportion of muscle fibers that exhibited stripes after the various stresses were applied. In control copepods, this percentage was variable between muscle regions (Fig 4a), from 100% (in m1 and m4) to 20% (in m6). The minor haline stress (H15; Fig 2b) had the most drastic effect on this percentage since stripes were no longer observed in 4 out of the 9 muscle regions studied. The impact of the mixed stress (M; 4°C, 0 psu) was less intense than the impact of the major haline stress (H0; 18°C, 0 psu), suggesting that the longer duration of the stress (less than 1 minute versus 12 minutes) contributed to the severity of its effects. In contrast, the thermal stress alone had either no effect (muscle regions m1 and m5) or induced an increase (muscle regions m2, m3 and m6 to m9) in the percentage of muscle fibers exhibiting stripes compared to the control (Fig 4a). We subsequently compared the distance between stripes in each region and for each stress (Fig 4b). This comparison was not always possible for the minor haline stress (H15) and for the region m6 in the control due to the disappearance of stripes in most muscle fibers. With thermal stress (T; Fig 2c), the average distance between stripes in each region (1.86 +/- 0.13 μm) was slightly smaller than in the control (2.06 +/- 0.11μm) except in m4 and m7 where this difference was not significant (p = 0.64 and 0.82 respectively for m4 and m7). For the major haline stress (H0) this average distance was longer than for the controls in the m1 and m3 regions (p<0.05). In contrast for the combined stressors, in each region except m7, the average distance between stripes (2.88 +/- 0.45 μm) was longer than in the control (p<0.05). The variability in the stripe distance was larger for the major haline and the mixed stressors. For the thermal stressor, the average distance between stripes in each region (1.86 +/- 0.13 μm) was significantly smaller than for the control (2.06 +/- 0.11μm) except in m4 and m7 where this difference was not significant (p = 0.64 and 0.82, respectively).

## Discussion

The effects of thermal or haline stressors on copepods have been observed at the levels of gene expression [14], protein expression [27] and enzymatic activity [28]. Our study showed, for the first time, the effects of these stressors on the muscular and cuticle structures of the copepod *Pseudodiaptomus marinus* adult females by using the CLSM tool. These rapid morphological effects were not related to the consumption of metabolic reserves by copepods. For another calanoid copepod *Eurytemora affinis*, nauplii became comatose after several hours but not minutes after their transfer from 15 to 0 psu [15]. We can conclude that even an euryhaline species like *E*. *affinis* that tolerates a high range of salinity variation in its estuarine habitat, is still not capable to manage an abrupt change of salinity. *P*. *marinus* is mainly a marine copepod with a narrower range of salinity habitats so the effect of a high haline stress is exacerbated. Nevertheless, both calanoid species *E*. *affinis* and *P*. *marinus*, seem not to becapable of osmoregulation when abruptly exposed to low water salinity of 0 psu.

In addition, major haline stress (H0) caused a crack in the cuticle (occurrence and number of cracks) which was not observed at low temperature in the combined stressor experiment (M). We explain this by the major pulse osmotic shock. The observation of material extruded from these cracks suggests that it results from an osmotic pressure that increases in the copepod body constrained by its cuticle. This region contains specific tissues that are specialized in ion transport and are responsible of osmoregulation in the calanoid copepod *E*. *affinis* [29]. This could explain that only this area was repeatedly affected (see Fig 2b and 2c). A similar haline shock was applied to the eggs of the euryhaline calanoid copepod *Acartia tonsa* after their transfer from 32 psu to 0 psu [30]. As a consequence, their volume immediately increased by 36%, but they never exploded but they also did not hatch [30]. In contrast, the embryos kept their volume and internal salinity constant and it was suggested that they were protected by their plasma membrane from abrupt salinity stresses [30]. This protection can be maintained in the first non-feeding naupliar stage as observed in the copepod *E*. *affinis* [15]. Our experiments show that this protection does not occur in adults of *P*. *marinus*.

In the present study, we used autofluorescence to describe muscle structure. This technique enabled us to visualize periodically ordered stripes parallel to each other in a subset of copepod muscle fibers. The autofluorescent signal coming from the muscular region revealed that both the percentage of the signal detection coming from muscle stripes and the distance between these stripes were affected by haline and thermal stresses. It was demonstrated before, that rapid changes in salinity affect the cellular metabolism of copepods, leading to modifications of individual swimming activity ([31]; [32]; [33]). Inhibitors of Acetylcholine Esterase (AChE) a common biomarker of activity commonly impair the swimming performance of aquatic animals [34]. AChE, is the primary cholinesterase in animals and mainly located in the neuromuscular junctions and in the chemical synapses. AChE activity in macro-crustacean muscle tissues (such as the common prawn *Palaemon serratus*, [35]) and in the pool of individuals in micro-crustacean (such as copepods, [28]; [36]) is correlated with their swimming activity. Despite these results suggesting the importance of muscles in the response of copepods to environmental stresses, no study was focusing so far on the subsequent modifications of copepod muscle structures.

The fluorescence signal coming from tissues originates from endogenous autofluorescent molecules such as nicotinamide adenine nucleotides (NAD(P)H) and oxidized flavoproteins (reviewed in [20]). Age, exercise, oxygen availability and muscle type that affect fiber composition would also change the autofluorescence of skeletal muscles. In addition, it has been shown that an autofluorescence signal arises upon binding of aldehyde-based fixatives to protein amine groups, allowing the visualization of sarcomeres in cardiomyocytes [37]. Glutaraldehyde is responsible for this effect in rodent skeletal muscles more than formaldehyde [38]. Formaldehyde (formalin) having only a weak effect on muscle tissue autofluorescence [39, 20]. Both flavins and NAD(P)H which could be excited by [350–600 nm] and [300–400 nm], respectively, are diffusing freely in the cytoplasm, and spatially ordered sarcomeric proteins contribute to muscle autofluorescence. The absence of a periodic autofluorescence pattern in a muscle fiber may, therefore, result either from sarcomere disassembly or from an accumulation of flavins or NAD(P)H with a continuous pattern. We propose that the drastic loss of fluorescence is coming from muscle stripes after flavin or NAD(P)H accumulation took place. This loss was moderate in case of minimal activity of copepods such as a mixed stress condition (M) during 12 minutes before getting comatose and it became important when copepods activity was decreasing such as during minor haline stress (H15) conditions. Interestingly, after the thermal stress (T), muscle regions showing this periodic autofluorescence pattern were more numerous than in control copepods (see Fig 4a). We suggest that this effect is a metabolic consequence of the slow motion of copepods during 75 minutes after their immersion in 0°C and 30 psu which is a condition where oxygen solubility is 40% higher than at 18°C and 30 psu.

Since exercise, stress and production of reactive oxygen metabolites induce proteolysis and thick filament disassembly [40, 41], further analysis of the effects of thermal and haline stresses on the overall structure of copepod muscle fibers is needed. Fluorescent phallotoxins have been used to stain F-actin and study muscle development in *Sicyonia ingensis* and *Artemia salina* larvae [42]. Yet the penetration of these dyes in copepod adults could be difficult considering the presence of an external cuticle as an impermeable barrier for most dyes. Alternatively, other nonlinear optic microscopy such as Second Harmonic Generation (SHG) or Coherent Anti Stokes Raman Spectroscopy (CARS) [43, 44] would allow the direct visualization of myosin molecules in thick filaments and of T-tubules independently of the NAD(P)H levels. Indeed, it was previously reported in skinned frog muscle fibers that the actin myosin spacing can be compressed osmotically by macromolecules such as Dextran [45, 46]. It was also observed that the distribution of stripes in cardiomyocytes is modified after a sudden uniaxial static strain, with weakly stained regions giving the appearance of gaps within a periodic pattern [47]. As previously demonstrated for *Xenopus laevis* [44], nonlinear optical microscopes as above could provide more precise information on sarcomere structure changes along *Pseudodiaptomus marinus* copepod myofibers subjected to thermal and haline stresses [48]. Besides its advantage, nonlinear optical microscopy is freely and easily available only to a relatively small number of scientists, while CLSM are present and accessible in most institutes today.

## Supporting Information

S1 Data The 5 measures of the distance between stripes taken from each region (m) for the 10 samples from each treatment (see Fig 3 legend). (XLSX)

S2 Data The percentage of stripes appearance in each treatment (see Fig 3 legend). We note by 1: appeared stripes, by 0disapeared stripes and by x: we don’t have information in this region.
(XLSX)

S3 Data The average of distance between stripes and stripes appearance in each treatment (see Fig 3 legend). (XLSX)
